# The Inflammatory Axis of *LncRNA Ftx/miR-382-5p*/NRG1 in the Differential Diagnosis and Prognosis of Multiple Sclerosis and Neuromyelitis Optica

**DOI:** 10.1007/s10753-025-02284-7

**Published:** 2025-04-07

**Authors:** Nadia Mangoud, Mohamed I. Hegazy, Shady Estfanous, Sahar A. Ali

**Affiliations:** 1 Center of Excellence, Helwan Structure Biology Research, Cairo, Egypt; 2 Biochemistry and Molecular Biology Department, Faculty of Pharmacy, Helwan University, Cairo, Egypt; 3 Neurology Department, Faculty of Medicine, Cairo University, Cairo, Egypt

**Keywords:** Neuromyelitis Optica (NMO), Multiple Sclerosis (MS), *lncRNA Ftx*, *miR-382-5p*, Neuregulin-1 (NRG1)

## Abstract

Multiple sclerosis (MS) and Neuromyelitis Optica (NMO) are immune-related CNS inflammatory diseases that often present with overlapping clinical symptoms, leading to frequent misdiagnosis, particularly in aquaporin-4 seronegative NMO patients. Identifying the underlying mechanisms of these diseases is critical for discovering biomarkers that enable timely diagnosis and effective treatment. This study included 252 participants, divided into four groups. **Group I** (Relapsing–Remitting MS: RRMS group), **Group II** (Secondary Progressive MS: SPMS group), **Group III** (NMO group), and **Group IV** (Healthy controls). Blood samples were collected from all participants to measure the expression levels of Neuregulin-1 (NRG1), *lncRNA Ftx,* and *miR-382-5p*. The results showed that NRG1 levels were significantly lower in the RRMS, SPMS, NMO groups compared to healthy controls, with the most pronounced reduction observed in NMO, suggesting NRG1 may serve as a potential biomarker for differentiating NMO from MS, especially in cases where traditional diagnostic criteria are inconclusive. *lncRNA Ftx*, a sponge for *miR-382-5p*, exhibited an opposite trend to miR-382-5p and was significantly downregulated in NMO compared to MS, SPMS, RRMS, and healthy controls, suggesting its potential as a promising biomarker. Our findings highlight the potential of NRG1, lncRNA Ftx, and miR-382-5p expression as diagnostic, screening, and prognostic biomarkers, as well as tools for the differential diagnosis of NMO and MS.

**Graphical Abstract:**

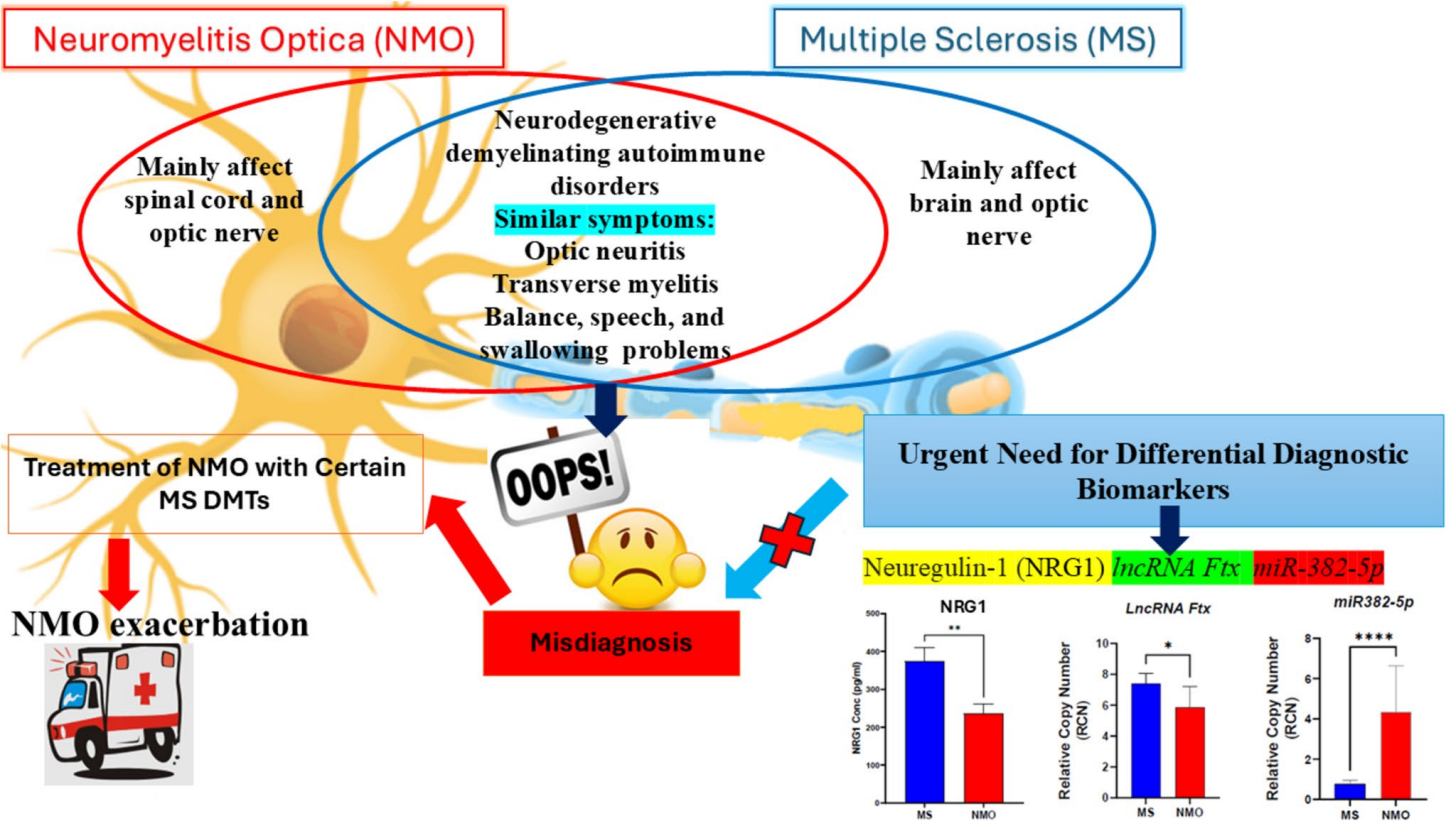

## Background

Multiple sclerosis (MS) is an inflammatory demyelinating disease of the central nervous system (CNS) with a variety of clinical presentations [1]. It is the most common non-traumatic disabling disease which affects early-mid aged adults (20–30 years) [2, 3].

Worldwide, MS cases have risen from 2.3 million in 2013 to 2.8 million in 2020, with a global prevalence of 35.9 per 100,000, affecting females more frequently than males [4]. In Egypt, prevalence tripled from 20 to 59.7 per 100,000 between 2013 and 2020, affecting 1 in 1,500 Egyptians [5].

The etiology of MS is unclear, but genetic and environmental factors, including EBV infection, smoking, vitamin D deficiency, and adolescent obesity are implicated [6, 7]. Immune cell infiltration and cytokine storms cause CNS gray and white matter inflammation and damage [8]. Symptoms varying with lesion location and include sensory, visual, motor, coordination impairments, spasticity, pain, fatigue, and cognitive deficits [9]. Most patients present a relapsing–remitting course (RRMS) with episodic relapses and partial or full recovery without progression. Within 8 years, one-third transition to secondary progressive MS (SPMS), marked by gradual disability progression [10]. MS is diagnosed using the 2017 McDonald Criteria, which combines clinical history, neurological examination, MRI of the brain and spinal cord, and laboratory findings (e.g., oligoclonal bands in the CSF). Although incurable, disease-modifying therapies (DMTs) reduce inflammation, myelin damage, and relapses [11].

Neuromyelitis Optica (NMO), an immune-mediated CNS demyelinating disease, mimics MS clinically, leading to frequent misdiagnosis. Unlike MS, NMO does not respond to MS disease-modifying drugs and may worsen with treatments like ß-interferons, glatiramer acetate, or fingolimod [12, 13]. NMO primarily affects the optic nerve and spinal cord and is marked by serum aquaporin-4 immunoglobulin G antibodies (AQP4-IgG). However, some NMO patients are AQP4-IgG seronegative or have undetermined results, highlighting the need for reliable serum or CSF biomarkers to aid early diagnosis and improve MS treatment outcomes [14–16].

Neuregulin-1 (NRG1) is a signaling protein crucial for CNS development, nerve growth, and myelination. The NRG1 beta 1 isoform, containing the epidermal growth factor (EGF) domain, plays a key role in reducing microglial inflammation and astrocyte reactivity in CNS injuries. Its dysregulation early in MS contributes to disease progression and severity. However, the expression of NRG1 in Egyptian MS patients and the mechanism behind its altered expression remain unknown and need further investigation [17–19].

Long non-coding RNAs (lncRNAs) are transcripts longer than 200 nucleotides that primarily function as competitive endogenous RNA (ceRNA) sponges. They sequester microRNAs (miRNAs), reducing miRNA activity and thereby regulating gene expression. While miRNAs (18–25 nucleotides) guide the RNA-induced silencing complex (RISC) to target mRNAs, leading to translation inhibition and degradation, lncRNAs can modulate this process, influencing protein levels and biological processes like immune responses and inflammation [20, 21].

LncRNA Ftx has been shown to reduce inflammation in conditions like spinal cord injury by upregulating NRG1β1 through miR-382-5p inhibition [22]. However, the roles of lncRNA Ftx and miR-382-5p in the pathogenesis and prognosis of Multiple Sclerosis (MS), as well as their potential in differentiating MS from Neuromyelitis Optica (NMO), are not yet fully understood.

## Subjects and Methods

### Study Participants

The study enrolled 252 participants, divided into four groups: Group I (RRMS) with 74 patients (12 males, 62 females, mean age 31.28 ± 1.09 years), Group II (SPMS) with 70 patients (25 males, 45 females, mean age 38.11 ± 1.31 years), Group III (NMO) with 38 patients (9 males, 29 females, mean age 36.26 ± 1.84 years), and Group IV (Healthy Controls) with 70 volunteers (26 males, 44 females, mean age 34.47 ± 1.46 years), matched for age, sex, and ethnicity with the MS and NMO groups, but RRMS and SPMS groups were not matched with controls. Clinically isolated syndrome (CIS) and primary progressive MS (PPMS) were not included due to their low prevalence. All MS patients included in this study received DMTs treatment. Some examples of those DMTs are Interferon beta-1a (Avonex®, Rebif®), Fingolimod (Gilenya®), Dimethyl fumarate (Tecfidera®), Natalizumab (Tysabri®), Ocrelizumab (Ocrevus®) and Teriflunomide (Aubagio®).

Healthy controls and MS and NMO patients were recruited from Cairo University Hospitals Blood Bank and the Kasr Al-Ainy Multiple Sclerosis Unit, Cairo, Egypt. MS diagnosis followed the 2017 McDonald Criteria, and NMO diagnosis, including seropositive and seronegative AQP4-IgG cases, adhered to International Consensus Guidelines. Participant demographics and clinical characteristics are detailed in Table 1. Written informed consent was obtained, and the study was approved by the scientific research ethics committee of Helwan University's Faculty of Pharmacy (March 2022, approval number 03H2022), in accordance with the Declaration of Helsinki [23, 24].

**Table 1:** Demographic and Clinical Characteristics of Study Groups

Parameters		Healthy N = 70	MS N = 144	NMO N = 38	*P*-value
Age (Min–Max) (Mean ± SEM) (Yrs)		(18 – 70) (34.5 ± 1.5)	(18—60) (35 ± 0.92)	(18—53) (36.3 ± 1.8)	> 0.05
Sex	Male N, % Female N, %	26 (37%) 44 (63%)	37 (26%) 107 (74%)	9 (24%) 29 (76%)	> 0.05
Disease Onset (Mean ± SEM) (Yrs)			(12—48) (26.04 ± 0.81)	(16—50) (31.15 ± 1.681)	< 0.01
OCB	Positive	0	139 (97%)	0	< 0.0001
	Negative	0	5 (3%)	29 (100%)	
	Not done	70 (100%)	0	9	
Aquaporin- 4	Positive	0	0	31 (81.5%)	< 0.0001
	Negative	0	44 (100%)	7 (18.5%)	
	Not done	70 (100%)	100 *	0	
EDSS (Mean ± SEM)		0	4.5 ± 0.18	4.6 ± 0.34	< 0.0001

**SEM**: Standard Error of Mean, **MS:** Multiple Sclerosis, **NMO**: Neuromyelitis Optica, **N**: Number, **OCB:** Oligoclonal Bands. **EDSS:** Expanded Disability Status Scale

Numerical data were done by independent t tests. Categorical data were compared by Chi square test (X^2^) P values < 0.05 are considered significant. * In Egypt, Aquaporin-4 testing is commonly performed; however, some MS cases are diagnosed based on MRI findings and the presence of oligoclonal bands (OCBs) in cerebrospinal fluid, making further Aquaporin-4 testing unnecessary. The percentages presented for **OCB** and **Aquaporin-4** reflect the proportion of patients who underwent the respective test, rather than the total cohort

### Laboratory Measurements

Venous blood samples were collected from all participants and processed into two aliquots. The first aliquot was transferred into serum-separating tubes, followed by centrifugation to isolate the serum, which was subsequently stored at −80 °C for the quantification of serum NRG1 levels. The second aliquot was collected in tubes containing 0.5 M EDTA and preserved at −20 °C for RNA extraction.

#### NRG1 Quantification

Serum NRG1 levels were quantified using a commercially available enzyme-linked immunosorbent assay (ELISA) kit (NOVA ELISA kit, Catalog No. In-Hu3368, INNOVA Biotech, Beijing, China) in accordance with the manufacturer’s protocol. This kit is designed to measure total NRG1 levels, encompassing multiple isoforms, but does not selectively quantify specific isoforms such as NRG1β1. The detection range and assay specificity were verified based on the manufacturer’s documentation.

#### Differential Expression of ncRNA by Polymerase Chain Reaction (PCR)

Total RNA was isolated from blood samples using the GENEzol™ TriRNA Pure Kit (Catalog No. GZX050, GZXD050, Geneaid, Taiwan). The RNA was then used for cDNA synthesis with the Thermo Scientific™ RevertAid™ First Strand cDNA Synthesis Kit (Catalog No. K1622, Thermo Fisher Scientific™, USA). Real-time quantitative PCR (qPCR) was performed using SensiFAST™ SYBR® No-ROX Kits (Bioline, United Kingdom), with the running conditions shown in Table 2. The relative expression levels of *miR-382-5p* and *lncRNA Ftx* were calculated as relative copy numbers (RCN) by determining the expression ratios of the target genes to their respective housekeeping genes (GAPDH for lncRNAs and U6 for miRNAs) using the equation: RCN = 2^(-ΔCt) × 100, where ΔCt is the difference between the Ct values of the target gene and reference gene. Primer sequences are listed in Table 3.

**Table 2:** Time–temperature protocol for qPCR reaction

Thermal condition	Temp	Time	No. of cycles
Holding activation step	95 °C	2 min	1 cycle
Denaturation	95 °C	10 s	45 cycles
Annealing and extension	58 °C	45 s	
Melting curve	65- 95 °C	90 s pre melt 5 s each step afterwards

**Table 3:** Sequences of the primers used in qPCR transcriptomics analysis (5’ to 3’)

Primers for PCR	Forward sequence	Reverse sequence
LncRNA FTX	GAATGTCCTTGTGAGGCAGTTG	TGGTCACTCACATGGATGATCTG
GAPDH	GGAGCGAGATCCCTCCAAAAT	GGCTGTTGTCATACTTCTCATGG
MiR-382-5p	GAGAAGTTGTTCGTGGTGGA	TGTCCGAATGATTCGTCATA
U 6	GCTTCGGCAGCACATATACTA	CGAATTTGCGTGTCATCCTTG

### Statistical Analysis

All statistical analysis were performed using GraphPad Prism software, version 9.0 (La Jolla, CA, USA). The expression levels of *miR-382-5p* and *lncRNA Ftx* were quantified using the comparative ΔCt method, while NRG1 expression was analyzed using one-way ANOVA, followed by Tukey’s post hoc test for multiple comparisons. Data distribution was assessed for normality using the Shapiro–Wilk test. Differences in biomarker expression between Neuromyelitis Optica (NMO) and Multiple Sclerosis (MS) patients were evaluated using an unpaired t-test. To further explore the impact of disease status and other variables on biomarker expression, a two-way ANOVA with Tukey’s post hoc test was applied. Correlations between the expression levels of *miR-382-5p*, *lncRNA Ftx*, and NRG1 were assessed using Spearman’s rank correlation coefficient. Receiver Operating Characteristic (ROC) curves were generated to evaluate the diagnostic and prognostic utility of these biomarkers. Statistical significance was defined as a p-value < 0.05.

## Results

### Serum NRG1 Levels as a Differential Diagnostic Biomarker in Multiple Sclerosis and Neuromyelitis Optica

**Fig. 1:**
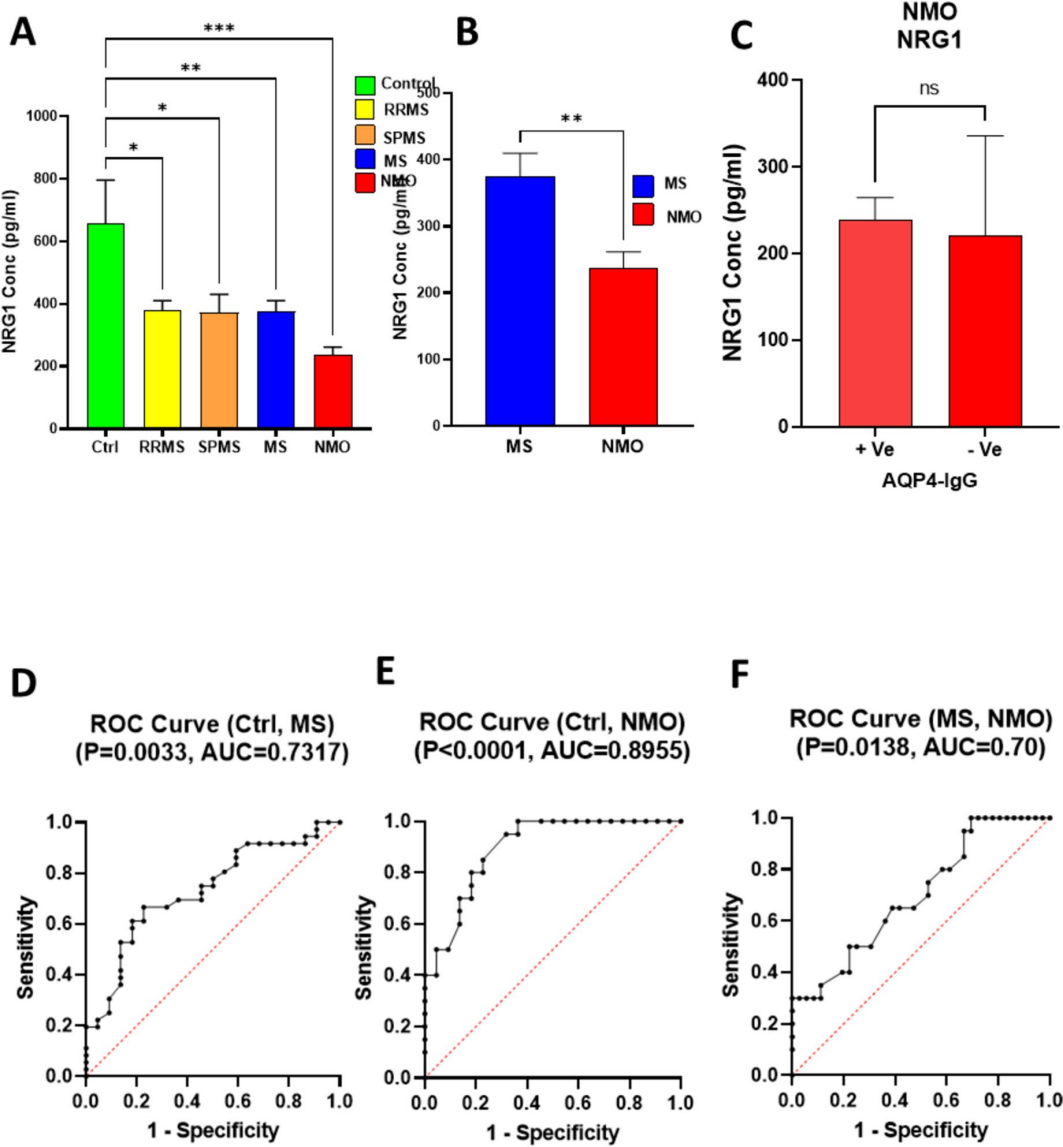
Analysis of NRG1 Concentration and Receiver Operating Characteristic (ROC) Curves for NRG1 Expression Levels in Different Studied Groups.** (A)** NRG1 concentration in different groups (Ctrl, RRMS, SPMS, MS, NMO). The data were analyzed using ANOVA with Tukey's correction for multiple comparisons. **(B)** Comparison of NRG1 concentration between NMO and MS, Unpaired t test is used. **(C)** Comparison of NRG1 concentration between NMO AQP4-IgG (+ ve) and NMO AQP4-IgG (-ve), Unpaired t test is used. ROC curve for Ctrl vs. Multiple Sclerosis (MS), **(E)** ROC curve for Ctrl vs. Neuromyelitis Optica (NMO), **(F)** ROC curve for MS vs. NMO. The area under the curve (AUC) values for each comparison are displayed above the respective curves, indicating the diagnostic accuracy of NRG1 expression for each condition. AUC values represent the ability of NRG1 to distinguish between the specified groups, with higher AUC values indicating better diagnostic performance. Statistical significance is indicated as follows: *p < 0.05, **p < 0.01, ***p < 0.00. All values are presented as mean ± SEM

Serum NRG1 levels were significantly reduced across all patient groups (RRMS, SPMS, MS, and NMO) compared to healthy controls, with the most significant decrease observed in NMO patients (Fig. 1A). Furthermore, NRG1 concentrations in NMO patients were significantly lower than those in MS patients (p = 0.0084, Fig. 1B). However, there was no significant difference in NRG1 concentration between NMO AQP4-IgG (+ ve) and NMO AQP4-IgG (-ve), p = 0.8356, Fig. 1C). ROC curve analysis demonstrated NRG1’s robust ability to distinguish both NMO and MS from healthy controls, with area under the curve (AUC) values of 0.73 and 0.89, respectively (Figs. 1D–E). Additionally, NRG1 showed potential in differentiating NMO from MS, as evidenced by an AUC of 0.70 (p = 0.0138, Fig. 1F).

### miR-382-5p expression as a Differential Diagnostic Biomarker in Multiple Sclerosis and Neuromyelitis Optica

*MiR-382-5p* is known to target NRG1 in spinal cord injury (SCI) [22]. However, its role in MS or and its potential as a differential marker between NMO and MS have not been fully explored. To address this, the expression of *miR-382-5p* was quantified across various groups: MS, NMO, RRMS, SPMS, and healthy controls counterpart.

**Fig. 2:**
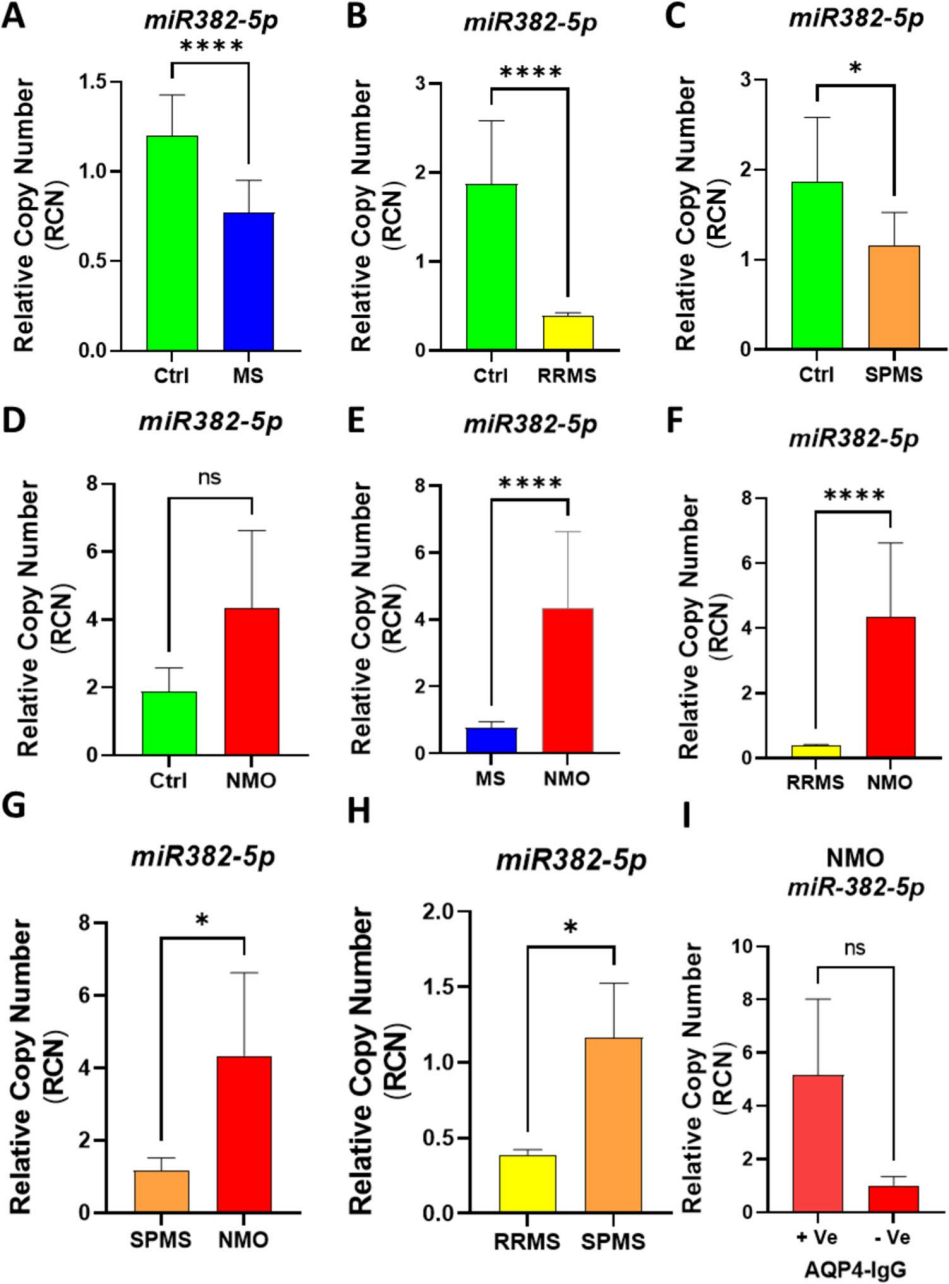
Analysis of *miR-382-5p* Relative Copy Number in Different Groups and Pairwise Comparisons.** (A-D).** Comparison of *miR-382-5p* relative copy number between control (Ctrl) and: **(A)** Multiple Sclerosis (MS), **(B)** Relapsing–Remitting Multiple Sclerosis (RRMS), **(C)** Secondary Progressive Multiple Sclerosis (SPMS), and **(D)** Neuromyelitis Optica (NMO). **(E–H)** Pairwise comparisons of *miR-324-3p* relative copy number between: **(E)** MS and NMO, **(F)** RRMS and NMO, **(G)** SPMS and NMO, **(H)** SPMS and RRMS, and **(I)** NMO AQP4-IgG (+ ve) and NMO AQP4-IgG (-ve). The Mann–Whitney U test was used for statistical analysis. Statistical significance is indicated as follows: *p < 0.05, **p < 0.01, ***p < 0.001. All values are presented as mean ± SEM

As illustrated in Fig. 2, *miR-382-5p* was significantly downregulated in MS, RRMS and SPMS compared to healthy controls, with p-values of (< 0.0001, < 0.0001 and 0.0130 respectively). On the other hand, *miR-382-5p* expression was significantly upregulated in NMO compared to MS, RRMS and SPMS (p < 0.0001, p < 0.0001 and p = 0.0101 respectively). Although elevated trends in *miR-382-5p* levels were observed in NMO compared to healthy controls, this difference did not achieve statistical significance (p = 0.5998). Additionally, there was no significant difference in *miR-382-5p* levels between NMO AQP4-IgG (+ ve) and NMO AQP4-IgG (-ve), p = 0.7090).

**Fig. 3:**
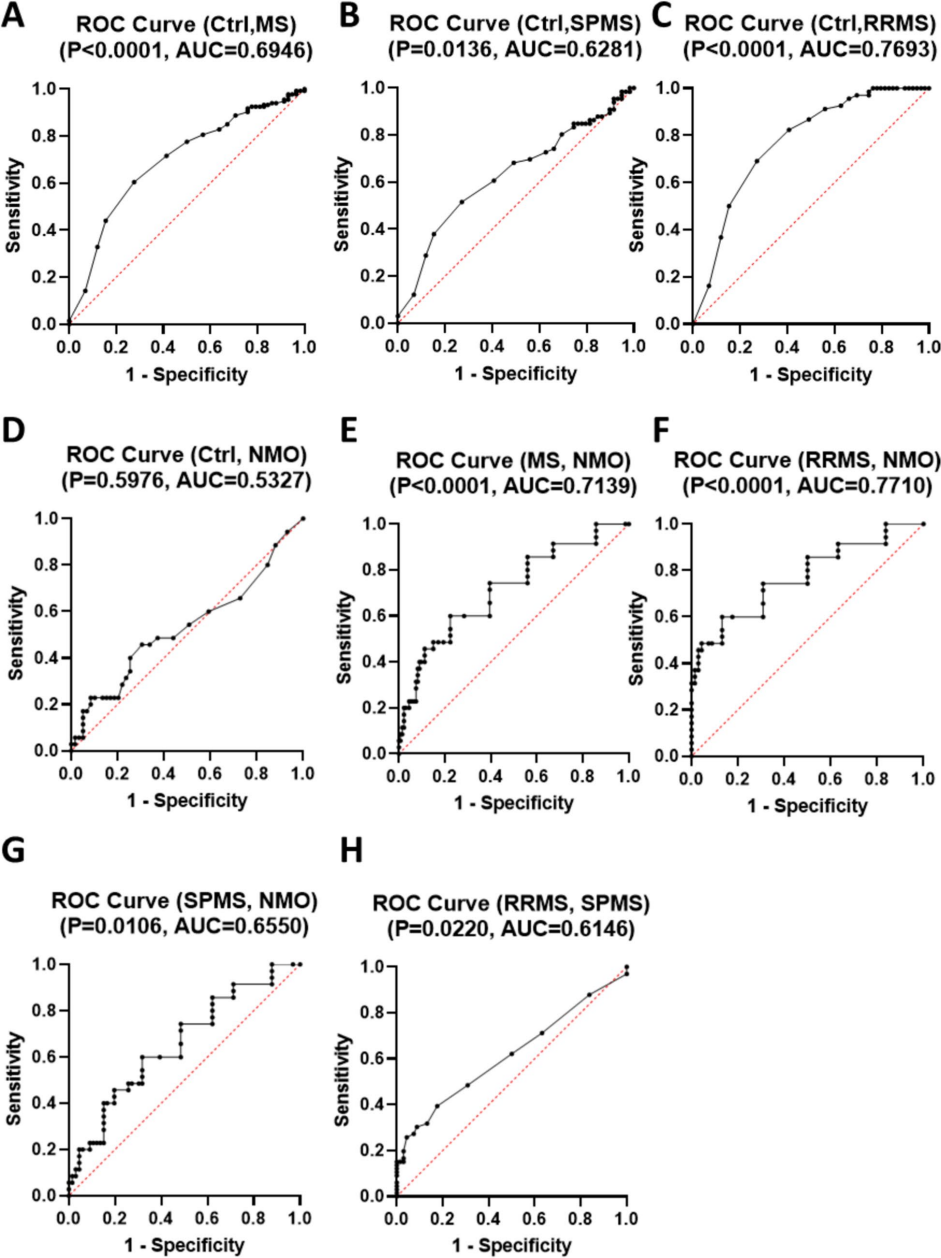
Receiver Operating Characteristic (ROC) Curves for *miR-382-5p* Relative Copy Number in Different Patient Groups. ROC curves illustrate the diagnostic performance of *miR-382-5p* relative copy number in distinguishing between control subjects (Ctrl) and various patient groups, as well as between different patient groups. **(A)** ROC curve for Ctrl vs. Multiple Sclerosis (MS), **(B)** Ctrl vs. Secondary Progressive Multiple Sclerosis (SPMS), **(C)** Ctrl vs. Relapsing–Remitting Multiple Sclerosis (RRMS), **(D)** Ctrl vs. Neuromyelitis Optica (NMO), **(E)** MS vs. NMO, **(F)** RRMS vs. NMO, **(G)** SPMS vs. NMO, and **(H)** RPMS vs. SPMS. The area under the curve (AUC) values for each comparison are displayed above the respective curves. Higher AUC values signify better diagnostic performance in distinguishing between the specified groups

To assess the potential of *miR-382-5p* as a biochemical biomarker, ROC curve analysis was performed. As illustrated in Fig. 3, *miR-382-5p* exhibited robust diagnostic utility for distinguishing between healthy controls and Multiple Sclerosis (MS) (p < 0.0001, AUC = 0.6946), Fig. (3A). However, its diagnostic performance for differentiating Neuromyelitis Optica (NMO) from healthy controls was limited (p = 0.5976, AUC = 0.5327), Fig. (3D). Notably, *miR-382-5p* was effective in distinguishing NMO from MS (p < 0.0001, AUC = 0.7139), Fig. (3E), Relapsing–Remitting MS (RRMS) (p < 0.0001, AUC = 0.7710), Fig. (3F), and Secondary Progressive MS (SPMS) (p = 0.0106, AUC = 0.6550), Fig. (3G). Additionally, *miR-382-5p* demonstrated potential as a prognostic marker by effectively differentiating RRMS from SPMS (p = 0.022, AUC = 0.6146), Fig. (3H).

### LncRNA Ftx Expression Levels as a Potential Biomarker in Multiple Sclerosis and Neuromyelitis Optica

**Fig. 4:**
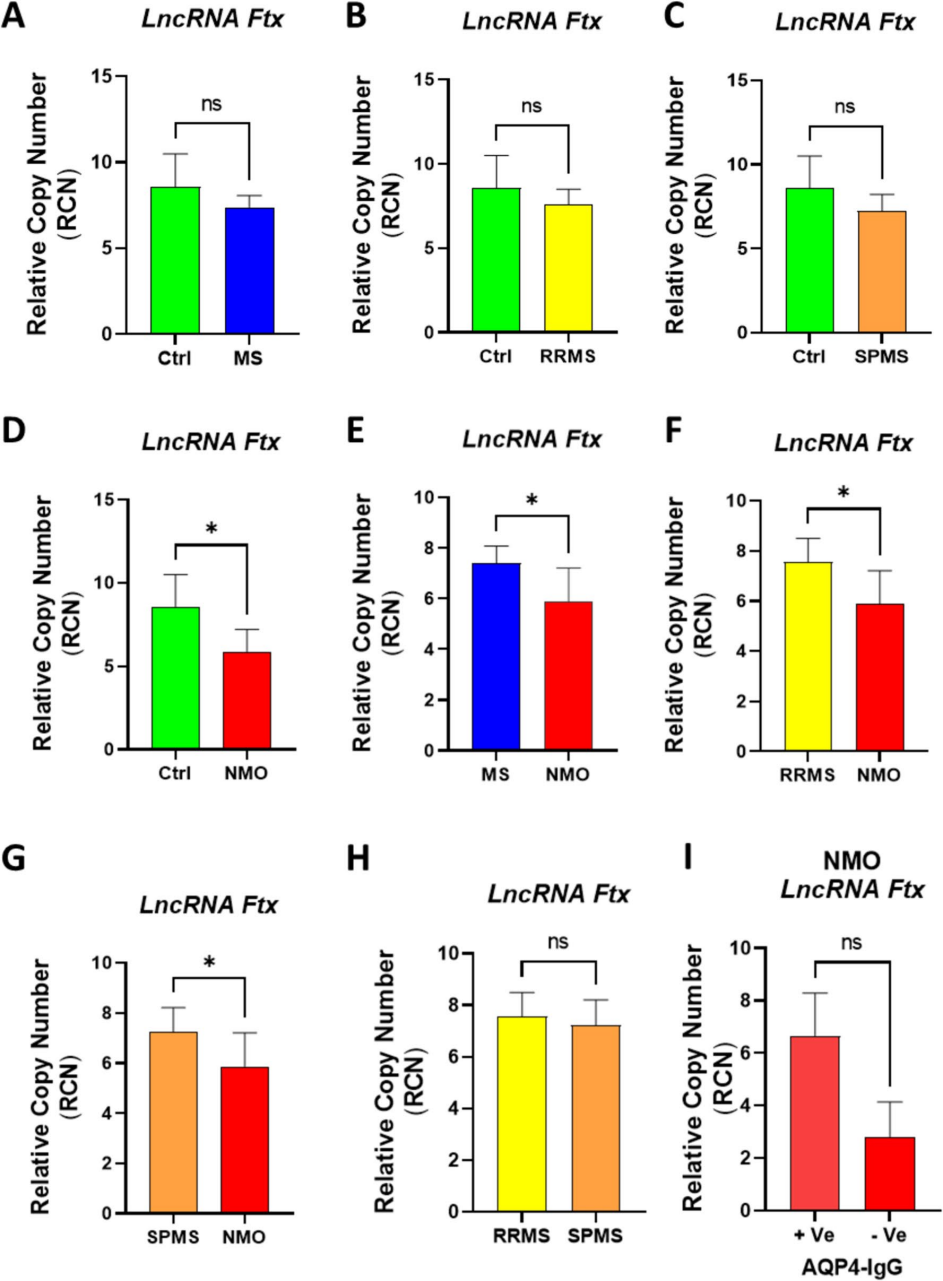
Analysis of *lncRNA Ftx* Relative Copy Number in Different Groups and Pairwise Comparisons.** (A-D)** Comparison of *lncRNA Ftx* relative copy number between control (Ctrl) and: (A) Multiple Sclerosis (MS), **(B)** Relapsing–Remitting Multiple Sclerosis (RRMS), **(C)** Secondary Progressive Multiple Sclerosis (SPMS), and **(D)** Neuromyelitis Optica (NMO). **(E–H)** Pairwise comparisons of lncRNA Ftx relative copy number between: **(E)** MS and NMO, **(F)** RRMS and NMO, **(G)** SPMS and NMO, **(H)** SPMS and RRMS, and **(I)** NMO AQP4-IgG (+ ve) and NMO AQP4-IgG (-ve).. The Mann–Whitney U test was used for statistical analysis. Statistical significance is indicated as follows: *p < 0.05, **p < 0.01, ***p < 0.001. All values are presented as mean ± SEM

*LncRNA Ftx*, which is known to act as a sponge for *miR-382-5p* and subsequently promote NRG1 expression, was assessed across MS, NMO, RRMS, SPMS, and healthy control groups counterpart. The goal was to evaluate *lncRNA Ftx*'s role in MS and its potential as a differential diagnostic marker for NMO. As depicted in Fig. 4 A-C, *lncRNA Ftx* expression showed no statistically significant differences between the control group and the various MS subtypes: MS (p = 0.4599), RRMS (p = 0.8499), and SPMS (p = 0.2705). Additionally, comparisons between RRMS and SPMS did not yield significant results (p = 0.4764). Figure 4I further indicated no significant difference in lncRNA Ftx levels between NMO AQP4-IgG (+ ve) and NMO AQP4-IgG (-ve) (p = 0.4269). Notably, in contrast to the relative upregulation of *miR-382-5p, lncRNA Ftx* level was significantly decreased in NMO compared to healthy controls, MS, RRMS, and SPMS (p = 0.0207, p = 0.0142, p = 0.0185, and p = 0.0356, respectively as shown in Fig. 4 D-F.

**Fig. 5:**
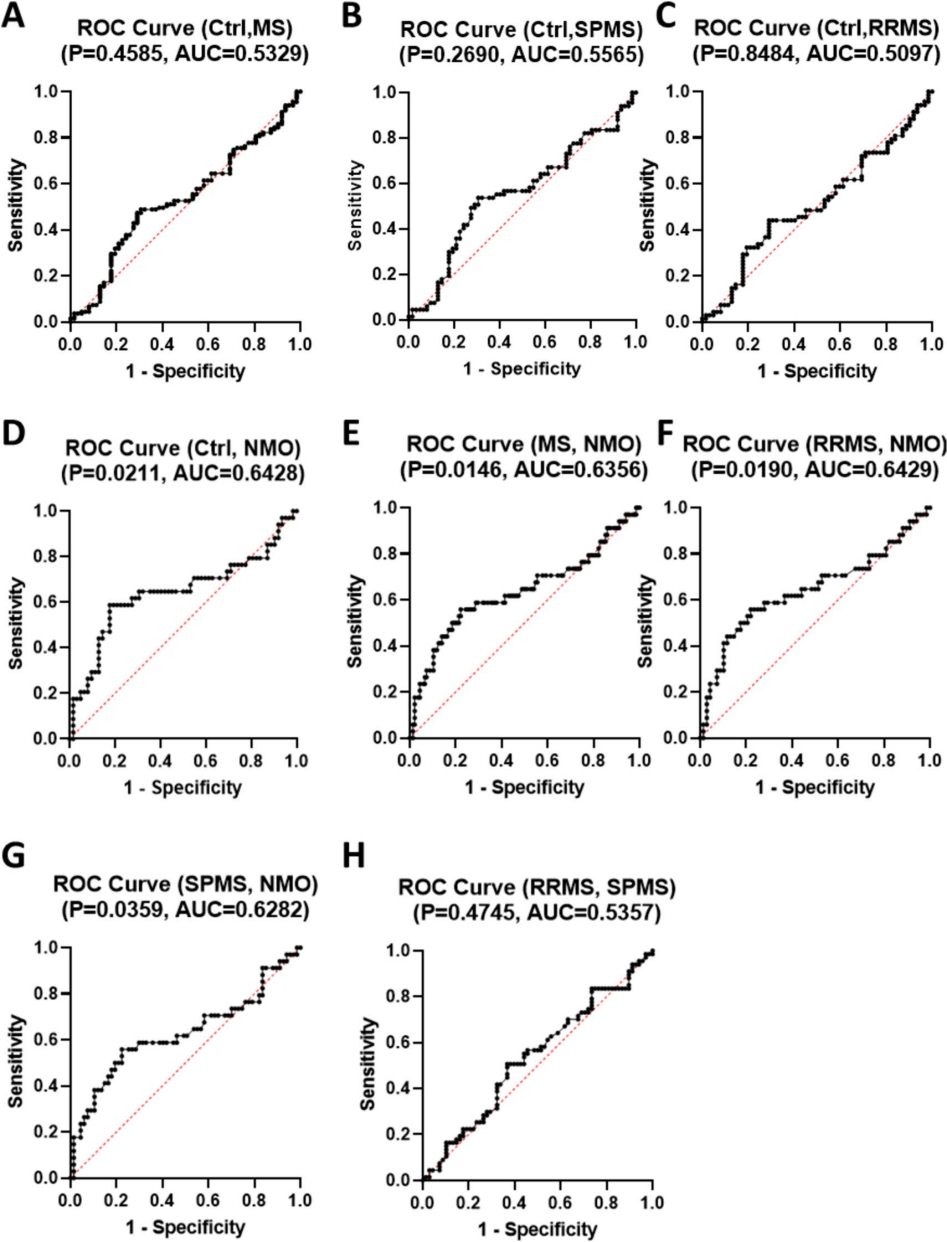
Receiver Operating Characteristic (ROC) Curves for *lncRNA Ftx* Relative Copy Number in Different Patient Groups. ROC curves illustrate the diagnostic performance of *lncRNA Ftx* relative copy number in distinguishing between control subjects (Ctrl) and various patient groups, as well as between different patient groups. **(A)** ROC curve for Ctrl vs. Multiple Sclerosis (MS), **(B)** Ctrl vs. Secondary Progressive Multiple Sclerosis (SPMS), **(C)** Ctrl vs. Relapsing–Remitting Multiple Sclerosis (RRMS), **(D)** Ctrl vs. Neuromyelitis Optica (NMO), **(E)** MS vs. NMO, **(F)** RRMS vs. NMO, **(G)** SPMS vs. NMO, and **(H)** RRMS vs. SPMS. The area under the curve (AUC) values for each comparison are displayed above the respective curves, indicating the diagnostic accuracy of *lncRNA Ftx* relative copy number for each condition. Higher AUC values signify better diagnostic performance in distinguishing between the specified groups

To evaluate the diagnostic and prognostic potential of *lncRNA Ftx*, ROC curve analysis was performed. As shown in Fig. 5, *lncRNA Ftx* demonstrated limited diagnostic utility for distinguishing between healthy controls and MS (p = 0.4585, AUC = 0.5329), SPMS (p = 0.2690, AUC = 0.5565), and RRMS (p = 0.8484, AUC = 0.5097). Additionally, comparisons between RRMS and SPMS (p = 0.4745, AUC = 0.5357) also indicated limited prognostic potential.

Remarkably, *lncRNA Ftx* exhibited notable diagnostic ability, effectively distinguishing NMO from healthy controls (p = 0.0211, AUC = 0.6428). Its diagnostic strength extends to differentiating NMO from MS (p = 0.0146, AUC = 0.6356), RRMS (p = 0.0190, AUC = 0.6429), and SPMS (p = 0.0359, AUC = 0.6282). These findings underscore *lncRNA Ftx*’s potential as a valuable biomarker in pinpointing NMO with impressive accuracy.

### Correlation between Different Non-Coding RNA and NRG1 Expression

**Fig. 6:**
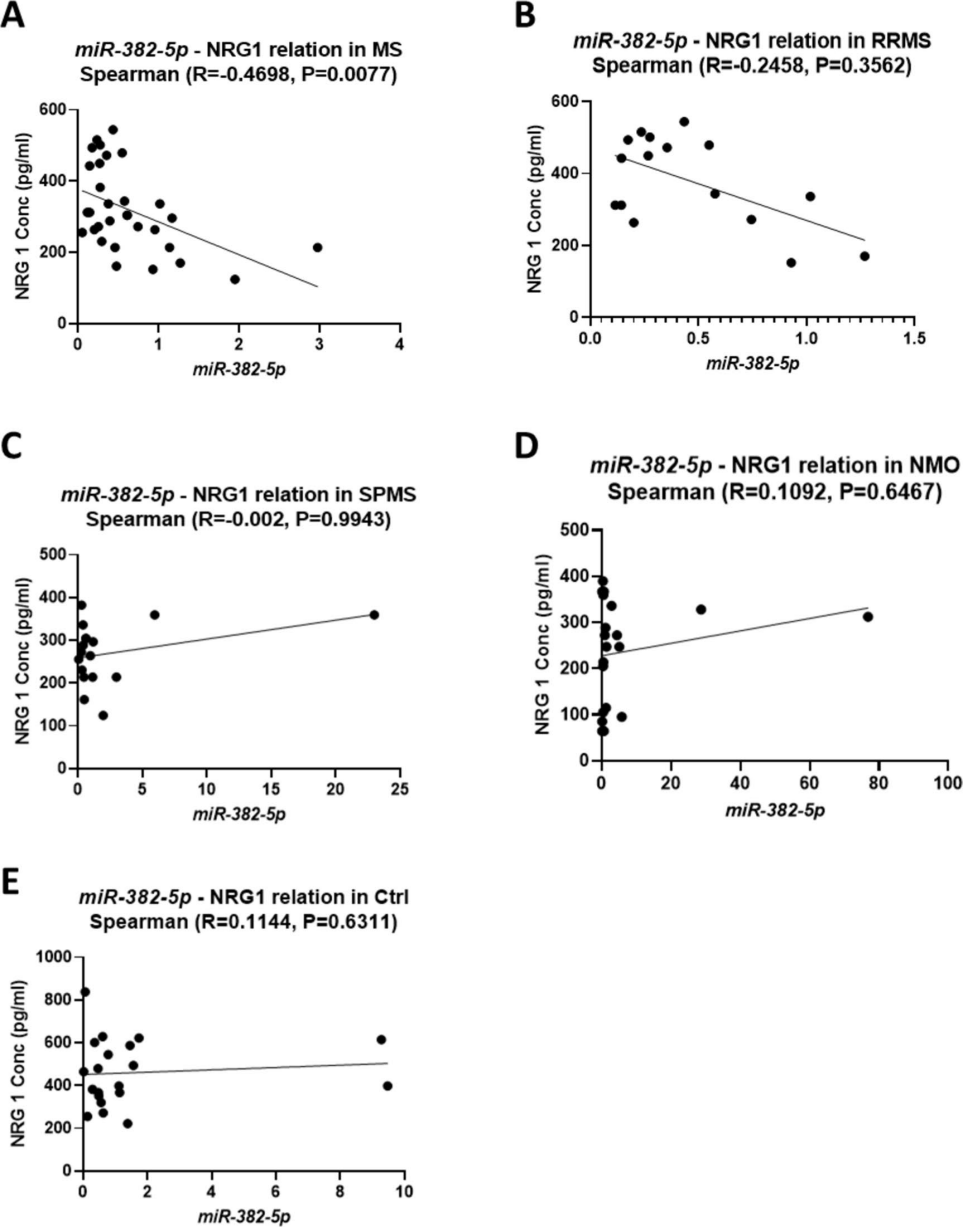
Spearman Correlation Between *miR-382-5p* and NRG1 Expression in Different Groups. Spearman correlation analysis between *miR-382-5p* relative copy number and NRG1 expression levels is presented for the following groups: **(A)** Multiple Sclerosis (MS), **(B)** Relapsing–Remitting Multiple Sclerosis (RRMS), **(C)** Secondary Progressive Multiple Sclerosis (SPMS), **(D)** Neuromyelitis Optica (NMO), and **(E)** Control (Ctrl). These figures illustrate the relationship between *miR-382-5p* and NRG1 in each respective group. The Spearman correlation coefficient (R) and p-value are indicated in each panel. A p-value below 0.05 is considered significant

Spearman correlation analysis was performed to assess the relationship between miR-382-5p and NRG1 expressions in various patient groups. As shown in Fig. 6, no significant correlation was observed between miR-382-5p and NRG1 expression in RRMS, SPMS, NMO, and control groups (R = −0.2458, P = 0.3562), (R = −0.002, P = 0.9943), (R = 0.1092, P = 0.6467), and (R = 0.1144, P = 0.6311), respectively. However, a significant negative correlation was found in the MS group (R = −0.4698, P = 0.0077), as shown in Fig. 6A.

**Fig. 7:**
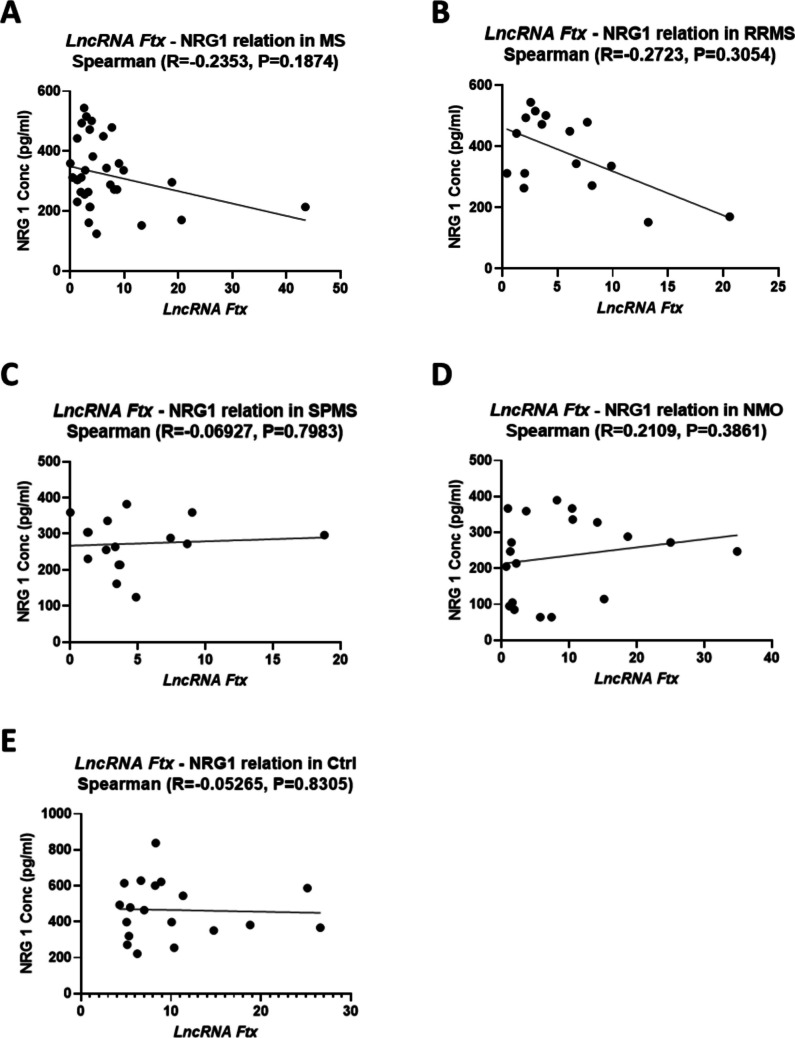
Spearman Correlation Between *lncRNA Ftx* and NRG1 Expression in Different Groups. Spearman correlation analysis between *lncRNA Ftx* relative copy number and NRG1 expression levels is presented for the following groups: **(A)** Multiple Sclerosis (MS), **(B)** Relapsing–Remitting Multiple Sclerosis (RRMS), **(C)** Secondary Progressive Multiple Sclerosis (SPMS), **(D)** Neuromyelitis Optica (NMO), and **(E)** Control (Ctrl). These figures illustrate the relationship between *lncRNA Ftx* and NRG1 in each respective group. The Spearman correlation coefficient (R) and p-value are indicated in each panel. A p-value below 0.05 is considered significant

In contrast, Fig. 7 shows no significant correlation between lncRNA Ftx and NRG1 expression across all groups. Specifically, no correlation was found in MS (R = −0.2353, P = 0.1874), RRMS (R = −0.2723, P = 0.3054), SPMS (R = −0.06927, P = 0.7983), NMO (R = 0.2109, P = 0.3861), or healthy controls (R = −0.05265, P = 0.8305).

**Fig. 8:**
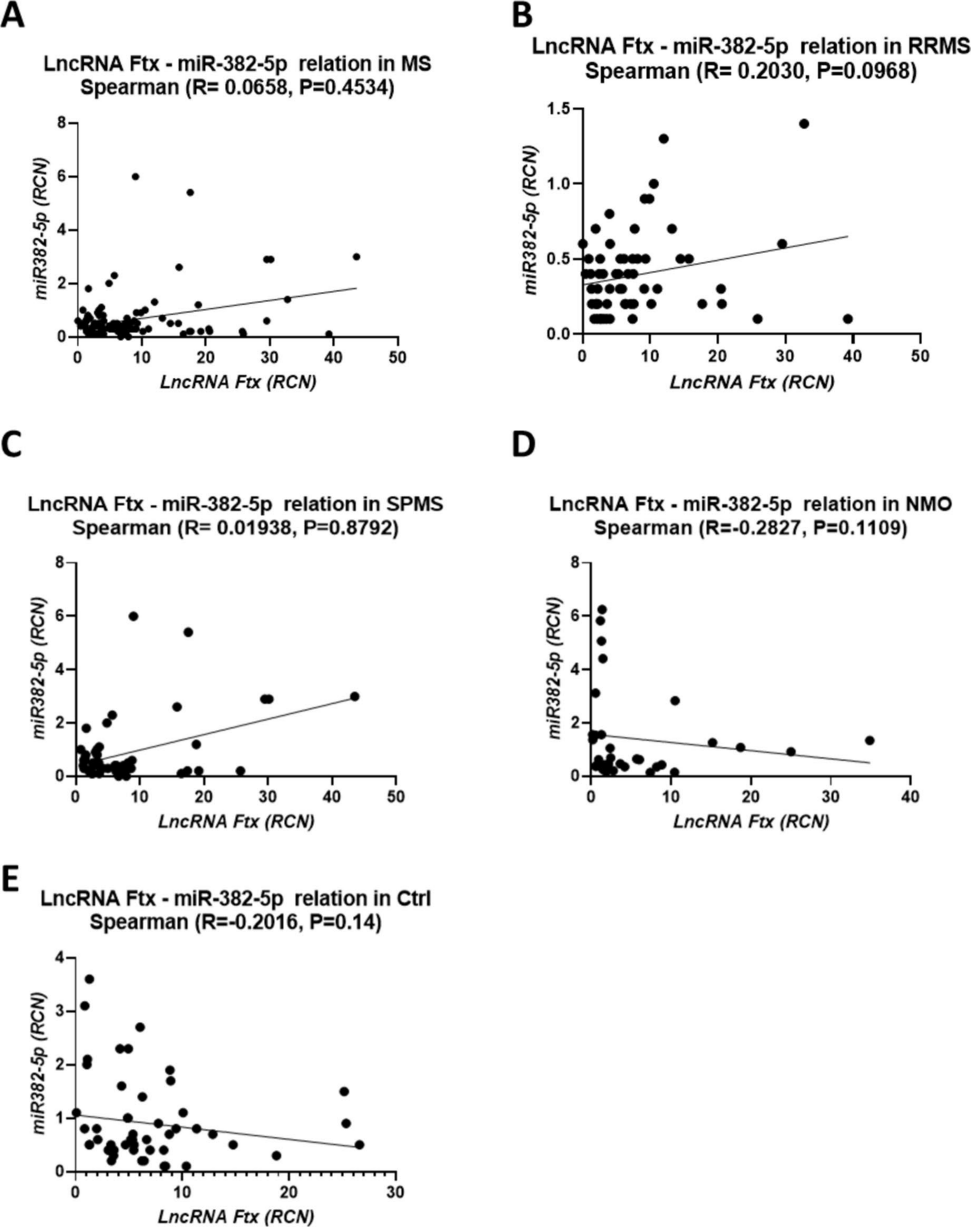
Spearman Correlation Between *lncRNA Ftx* and *miR-382-5p* Relative Copy Number in Different Groups. Spearman correlation analysis between *lncRNA Ftx* relative copy number and *miR-382-5p* relative copy number is presented for the following groups: **(A)** Multiple Sclerosis (MS), **(B)** Relapsing–Remitting Multiple Sclerosis (RRMS), **(C)** Secondary Progressive Multiple Sclerosis (SPMS), **(D)** Neuromyelitis Optica (NMO), and **(E)** Control (Ctrl). The panels illustrate the relationship between *lncRNA Ftx* and *miR-382-5p* RCN in each respective group. The Spearman correlation coefficient (R) and p-value are indicated in each panel. A p-value below 0.05 is considered significant

Figure 8 further demonstrates the absence of significant correlations between lncRNA Ftx and miR-382-5p expression across all groups. In MS, the correlation was R = 0.0658 (P = 0.4534), in NMO R = −0.2827 (P = 0.1109), in SPMS R = −0.01938 (P = 0.8792), in RRMS R = 0.2030 (P = 0.0968), and in healthy controls R = −0.2016 (P = 0.14). These results suggest complex and independent regulatory interactions between non-coding RNAs in these diseases.

### Diagnostic Performance of Biomarkers for MS and NMO Differentiation

The diagnostic performance of NRG1, miR-382-5p, and lncRNA Ftx for distinguishing MS and NMO from healthy controls, as well as between MS and NMO, is summarized in Supplementary Table 1. NRG1 concentration < 1021 pg/ml showed 97% sensitivity and 9% specificity for screening MS versus healthy controls, while < 226.6 pg/ml yielded high specificity (95%) but low sensitivity (19%) for diagnosis. For NMO, NRG1 at < 386.2 pg/ml showed 95% sensitivity and 63% specificity in screening, with < 234.9 pg/ml achieving 95% specificity and 40% sensitivity for diagnosis. miR-382-5p RCN performed well for screening MS versus healthy controls, with a cut-off < 16.25 showing 99% sensitivity but 0% specificity. For NMO, screening at > 0.14 showed 97% sensitivity and 14% specificity, while the diagnosis of MS was best at < 0.15 (14% sensitivity, 93% specificity) and NMO at > 14.63 (57% sensitivity, 99% specificity). LncRNA Ftx RCN demonstrated perfect sensitivity (100%) for screening NMO versus healthy controls at < 76.49, but with very low specificity (2%). For MS versus NMO diagnosis, < 0.09 showed 0% sensitivity but 98% specificity. The average values across both groups indicated moderate diagnostic performance, with 56% sensitivity and 75% specificity for MS/NMO differentiation.

In summary, the results demonstrate that while individual biomarkers such as NRG1, *miR-382-5p*, and *lncRNA Ftx* exhibit varying levels of sensitivity and specificity, their combined diagnostic potential could offer valuable insights for distinguishing between MS, NMO, and healthy controls. Further exploration of these biomarkers in larger cohorts and their role in disease progression may enhance their clinical utility for differential diagnosis and prognosis in autoimmune neuroinflammatory disorders.

## Discussion

This study investigates the diagnostic and prognostic potential of non-coding RNAs, specifically *miR-382-5p* and *lncRNA Ftx*, besides NRG1 protein expression, in distinguishing MS, NMO, and healthy controls. The patients in this study were under treatment. B cell therapy has been administered to patients with SPMS, and PRMS. B cell therapy, also known as B cell ablative therapy, targets cells called B cells that damage the nerves in the brain and spinal cord. Our findings highlight the differential expression of these biomarkers across groups*. miR-382-5p* was significantly upregulated in NMO compared to MS subtypes and healthy controls, while *lncRNA* Ftx exhibited a marked decrease in NMO. However, it demonstrated the potential to distinguish both MS and NMO from healthy controls. indicating its potential as a biomarker for identifying these conditions. The study further demonstrated that miR-382-5p plays a role in distinguishing NMO from MS, with robust diagnostic performance in differentiating MS from healthy controls. However, its ability to differentiate NMO from healthy controls was limited*. LncRNA Ftx*, while showing potential in distinguishing NMO from both MS and healthy controls, had limited diagnostic and prognostic utility in MS subtypes, with its expression levels being particularly low in NMO. Correlation analysis revealed complex, independent regulatory interactions between these biomarkers, with miR-382-5p and NRG1 showing significant negative correlation specifically in the MS group.

The findings underscore the potential of miR-382-5p and *lncRNA Ftx* as biomarkers for distinguishing NMO from MS, but their diagnostic utility is not without limitations. While *miR-382-5p* demonstrated high sensitivity for distinguishing MS from healthy controls, its specificity for NMO was suboptimal. *LncRNA Ftx*, although demonstrating high specificity in certain comparisons, exhibited low sensitivity for both MS and NMO. The results also suggest that combining these biomarkers could enhance diagnostic accuracy, though further studies with larger cohorts are necessary to validate their clinical utility.

Our findings partially align with those of Kataria et al., who demonstrated dysregulation of NRG1, particularly the NRG1β1 isoform, in MS pathology. However, we observed significant differences in total NRG1 levels between healthy controls and patients with NMO, MS (RRMS and SPMS), while Kataria et al. did not report such differences between MS patients and healthy controls. These discrepancies may stem from differences in the isoforms measured (total NRG1 vs. NRG1β1), study populations, and sample sizes, as well as methodological variations in quantification and processing techniques [19].

Additionally, prior studies have highlighted the protective, anti-inflammatory, and proliferative roles of NRG1 in neuroinflammatory diseases and various conditions, these include traumatic injury [25], LPC-induced focal demyelinating lesions [18], schizophrenia [26], psychosis [27], Stroke [28], autism [29], symptomatic epilepsy [30] and Alzheimer's disease [31], also, different tumors including lung cancer [32], breast cancer [33], esophageal squamous cell carcinoma [34]. These findings suggest that NRG1 is not only a marker of disease progression but also a potential therapeutic target for modulating inflammatory and neurodegenerative processes, as demonstrated in both MS and NMO by our current study.

Previous efforts to identify serum biomarkers for distinguishing NMO from MS have proposed candidates such as VEGF, MPIF-1, and NrCAM. However, these studies have largely relied on the presence of AQP4-IgG titers to confirm NMO diagnosis [35]. Additionally, analyses of cytokines, chemokines, and soluble receptors were conducted in MS patients and AQP4-IgG-positive NMO patients, but not in AQP4-IgG-negative NMO patients [36]. This approach is limited by the fact that a significant number of NMO patients are seronegative for AQP4-IgG, increasing the risk of false-negative results [35]. Our study advances this field by focusing on serum NRG1 levels in both AQP4-IgG-positive and negative NMO patients. We observed significantly lower NRG1 concentrations in NMO patients compared to MS patients, suggesting that NRG1 may serve as a more reliable biomarker for distinguishing between these conditions. This could address the limitations of current diagnostic approaches and improve diagnostic precision. Moreover, NRG1’s potential as a biomarker could pave the way for more personalized treatment strategies and highlight its promise as a therapeutic target in both MS and NMO. However, the efficacy of NRG1 as a prognostic biomarker was limited, particularly for distinguishing between RRMS and SPMS.

Non-coding RNAs, including lncRNAs and miRNAs, can both positively and negatively, directly and indirectly, influence protein expression, such as NRG1, positioning them as key players in the pathogenesis of neurodegenerative diseases, including MS [37]. For instance, numerous *miRNAs* have been identified as significantly contributing to the pathogenesis of MS and NMO, including *hsa-miR-16*, *hsa-miR-15a*, *hsa-miR-124*, *hsa-miR-26b*, *hsa-miR-181*, *hsa-miR-633*, *hsa-miR-155*, and *hsa-miR-129–2-3p* [38–42].

Notably, *miR-382-5p* has also been shown to target NRG1 in spinal cord injury [22] and its involvement extends to the pathogenesis of various neurological disorders, including migraine [43], schizophrenia [44], and cerebral ischemia–reperfusion injury [45]. Beyond its role in CNS disorders such as spinal cord injury [22, 46], glioma cell tumorigenesis [47], and depression [48], *miR-382-5p* also impacts other conditions, including liver regeneration, inflammation and various cancers [49–53].

Our study highlights the potential of *miR-382-5p* as a valuable biomarker for distinguishing between MS subtypes and healthy controls, with significant downregulation observed in patients with MS, SPMS, and RRMS. This downregulation suggests that *miR-382-5p* could serve as an effective marker for accurately differentiating MS subtypes from healthy individuals.

Notably, *miR-382-5p* also demonstrated the ability to differentiate between NMO and other MS subtypes, with significant upregulation in the NMO group compared to various MS subtypes. This upregulation underscores its potential as a diagnostic marker for NMO. The contrasting expression patterns—overexpression of *miR-382-5p* in NMO and downregulation in MS—may indicate distinct roles for this miRNA in the pathogenesis of MS and NMO, highlighting the complexity of these disorders. Furthermore, *miR-382-5p* was significantly different between RRMS and SPMS, so it could also serve as a prognostic marker for progression to SPMS in MS patients, further enhancing its diagnostic and prognostic utility.

Long non-coding RNAs (lncRNAs), which can act as competitive endogenous RNAs (ceRNAs) or enhance miRNA activity, play a crucial role in modulating gene expression. This dual functionality likely contributes to the complex regulatory mechanisms observed in MS and NMO pathogenesis, particularly in relation to miRNA interactions [54, 55]. Several lncRNAs have been implicated in the pathogenesis of MS and NMO, including *TUG, LRRC75A-AS1, LINC00293, BDNF-AS, GAS5, lncDDIT4,* and *MALAT1* [56, 57].

*LncRNA Ftx* has been implicated in various disorders, including neurological conditions such as spinal cord injury (SCI) [22] and stroke [58]. Beyond the CNS, it plays a role in malignancies including gastric cancer [59], colorectal cancer [60], glioma [61], and pancreatic cancer [62]. Furthermore, it is involved in non-cancerous diseases like asthma [63] and endometriosis [64]. Notably, *LncRNA Ftx* has also been shown to target *miR-382-5p* linking it to regulatory pathways relevant to both neurological injury and inflammation [22]. Moreover, *lncRNA Ftx* has been implicated in the pathogenesis of various neurological disorders, including epilepsy [65], cerebral ischemia–reperfusion injury [66], and autism [67].

In our study, although *LncRNA Ftx* showed no significant diagnostic or prognostic value in distinguishing between healthy controls and other MS subtypes, it notably demonstrated ability to differentiate NMO from both healthy controls and MS subtypes. The significant downregulation observed in the NMO group suggests its potential as a differential diagnostic marker for NMO. However, *LncRNA Ftx* did not exhibit prognostic utility for predicting progression to SPMS in MS patients.

Interestingly, the direct inverse relationship between NRG1 and *miR-382-5p* expression, previously observed in spinal cord injury (SCI) [22], was seen in MS group in our study but not reach significant level in Spearman correlation analysis in the rest of the groups. Additionally, the expected direct positive relationship between NRG1 and *lncRNA Ftx* expression, previously identified in spinal cord injury (SCI) [22], was not replicated in our study, possibly need larger sample size These results emphasize the complex regulatory dynamics of *non-coding RNAs* in the pathogenesis of MS and NMO, further highlighting the intricate molecular mechanisms.

The expected inverse relationship between lncRNA Ftx and miR-382-5p was not observed in any of the studied groups, highlighting the complex and multifaceted regulatory mechanisms underlying these diseases. Similar interactions have been reported in other conditions, such as oral squamous cell carcinoma [68], gastric cancer [69], and lung carcinoma [70], where non-coding RNAs and their interactions influence various molecular processes. While some correlations in our study were statistically significant, the interpretation of these results should consider the potential for non-coding RNAs to impact each other's regulatory roles.

Recent studies have highlighted the potential of various serum biomarkers for diagnosing and prognosticating Multiple Sclerosis (MS). Notably, an autoantibody signature has been proposed as a predictive biomarker for MS, demonstrating strong potential for early detection and diagnosis [71]. Additionally, the study by Ovchinnikova et al. [72] suggested that the high heterogeneity of cross-reactive immunoglobulins in MS supports the combination of B-cell epitopes for diagnostic purposes. Further research has combined serum markers with optical coherence tomography angiography to improve diagnostic accuracy for both MS and Neuromyelitis Optica (NMO) [73]. Moreover, serum markers such as GFAP and NfL levels have been shown to differentiate between subsequent progression and disease activity in patients with progressive MS [74]. These findings, along with our current study on the inflammatory axis of LncRNA Ftx/miR-382-5p/NRG1, emphasize the growing potential of biomarkers in improving diagnostic precision and differentiating MS from other conditions, such as NMO.

This study has several limitations. Its retrospective design and challenges within the Egyptian healthcare system mean that patients may have previously received corticosteroids, potentially limiting the analysis of treatment-naïve MS patients. The inclusion of potentially misdiagnosed cases adds complexity to the findings, and the absence of treatment-naïve MS and NMO patients precluded analysis of the lncRNA Ftx/miR-382-5p/NRG1 axis across treatment groups. The focus on total NRG1 rather than isoform-specific analysis, due to logistical constraints, limited deeper insights into the distinct roles of NRG1 and NRG1β1. Additionally, the small, single-center cohort, lack of regular frequent cerebrospinal fluid samplying, and uncollected Epstein-Barr virus (EBV) status further restrict generalizability. Future studies with larger, more diverse cohorts, inclusion of treatment-naïve patients, and isoform-specific analyses are essential to validate and expand upon these findings. Furthermore, previous studies have shown that different DMTs variably impact immune cell subsets, including T cells and IL-4 expression, with some having no effect and others modulating specific immune pathways. While our study did not specifically analyze the effects of individual DMTs on biomarker expression, their potential influence should be considered when interpreting our results. Future studies with larger, more diverse cohorts, inclusion of treatment-naïve patients, and isoform-specific analyses are essential to validate and expand upon these findings. Additionally, further research should explore how DMTs influence the regulatory networks of lncRNAs, miRNAs, and cytokines in MS pathogenesis.

Our study highlights the potential of NRG1, *miR-382-5p* and *lncRNA Ftx* as transformative biomarkers in distinguishing between MS and NMO, offering new avenues for more precise diagnostics and personalized treatments. The significant dysregulation of NRG1 in MS, along with its differential levels between MS and NMO, underscores its importance in advancing neuroimmunology. While we have made substantial progress, continued research is essential to fully leverage these insights for improved therapeutic strategies and patient outcomes.

## Data Availability

No datasets were generated or analysed during the current study.
